# Free Flaps for Advanced Oral Cancer in the “Older Old” and “Oldest Old”: A Retrospective Multi-Institutional Study

**DOI:** 10.3389/fonc.2019.00604

**Published:** 2019-07-03

**Authors:** Alberto Grammatica, Cesare Piazza, Raul Pellini, Nausica Montalto, Davide Lancini, Alperen Vural, Francesco Barbara, Marco Ferrari, Piero Nicolai

**Affiliations:** ^1^Department of Otorhinolaryngology—Head Neck Surgery, University of Brescia, Brescia, Italy; ^2^Department of Otorhinolaryngology, Maxillofacial and Thyroid Surgery, Fondazione IRCCS, National Cancer Institute of Milan, University of Milan, Milan, Italy; ^3^Department of Otolaryngology and Head and Neck Surgery, IRCCS National Cancer Institute, Rome, Italy; ^4^Department of Otorhinolaryngology—Head Neck Surgery, University of Bari, Bari, Italy

**Keywords:** oral cancer, elderly, free flaps, advanced age, medical complications, surgical complications, ACE27, ASA

## Abstract

**Introduction:** Surgery followed by adjuvant therapy represents the most adequate treatment for advanced oral squamous cell carcinoma (OSCC). Free flaps are considered the best reconstructive option after major oral surgery. In the last decades, OSCC has increased in the elderly due to an augmented life span. The aim of this work is to evaluate the feasibility of microvascular surgery in patients older than 75 years, focusing on clinical and surgical prognosticators.

**Methods:** “Older old” (aged ≥ 75) and “oldest old” (>85) patients who underwent microvascular reconstruction for OSCC from 2002 to 2018 were retrospectively evaluated in three referral Head and Neck Departments. Demographic, clinical, and surgical data were collected and analyzed. Pre-operative assessment was performed by ASA and ACE-27 scores. Complications were grouped as medical or surgical, and major or minor according to the Clavien-Dindo scale.

**Results:** Eighty-four patients (72 “older old” and 12 “oldest old”) were treated with a free flap success rate of 94.1%. Thirty-seven (44.7%) and nine (10.7%) patients had minor and major medical complications, respectively; 18 (21.4%) and 17 (20.2%) had minor and major surgical complications, respectively. Twenty-one (25%) patients had both medical and surgical complications (with a statistically significant association, *p* = 0.018). Overall, 52 (61.9%) patients had at least one complication: ASA score, diabetes mellitus, and duration of general anesthesia (DGA) significantly impacted the complication rate at multivariate analysis.

**Conclusion:** Our data confirm the feasibility of free flaps for OSCC reconstruction in appropriately selected elderly patients. Pre-operative assessment and aggressive management of glycemia in patients with diabetes is mandatory. DGA should be reduced as much as possible to prevent post-surgical complications. Comprehensive geriatric assessment is of paramount importance in this subset of patients.

## Introduction

While therapeutic and survival improvements have been recently reported for many head and neck cancers (HNC), oral squamous cell carcinoma (OSCC) management has not demonstrated substantial changes in the last decades and still remains challenging for both patients and surgeons. The National Comprehensive Cancer Network guidelines for advanced (Stages III-IV) OSCC indicate surgery with adjuvant treatments as the mainstay, whereas upfront radiotherapy (RT) or combined chemoradiation (CRT) should be considered when surgery is not feasible (1).

Epidemiological data projections from GLOBOCAN 2012 report that, among the 500,000 newly diagnosed cases in 2035, almost a third will occur in the elderly (>65 year-old) (2). It is a fact, indeed, that a major demographic shift is on the way, especially in industrialized countries, since life expectancy is increasing as the result of better prevention, medical, and surgical treatment, and improvements in overall social and economic status. This has led to a substantial increase of the proportion of older individuals in relation to the general population and, thus, to a redefinition of a proper cut-off for designation of the elderly. The US National Institute of Aging establishes the lower limit of elderly at 65 years and subdivides this cohort into three subgroups: (1) 65–74 years defined as the “young old;” (2) 75–84 years or the “older old;” and (3) 85 years or more as the “oldest old” (3).

In a retrospective review on HNC, 12% of patients were found to be older than 70 years (4). Moreover, data from different European centers have shown how 6–32% of HNC patients are nowadays diagnosed between 70 and 75 years of age (5, 6). According to these data, it is conceivable that, in the next 20 years, major surgical procedures requiring composite resections and some kind of reconstruction for OSCC will be even more frequently addressed in an increasingly elderly population, possibly with a more advanced comorbidity status. In fact, since their introduction 30 years ago, microvascular flaps are increasingly considered the first (and sometimes the only) choice for reconstruction of complex head and neck defects, especially in the oral cavity, due to their unique possibility to tailor the best tissue to be harvested according to the surgical defect created, reaching a success rate of 90–98% (7–9).

The aim of this study is to evaluate the feasibility and outcomes of free flaps reconstruction during major surgical procedures for OSCC in patients aged 75 years or more, focusing on factors that can impact on the rate of complications.

## Materials and Methods

This multi-institutional retrospective study was conducted in three Italian referral Head and Neck Departments: (1) Otorhinolaryngology—Head and Neck Surgery of the University of Brescia; (2) Otorhinolaryngology—Head and Neck Surgery of the National Cancer Institute “Regina Elena” in Rome, and (3) Otorhinolaryngology, Maxillofacial, and Thyroid Surgery of the National Cancer Institute in Milan. It included OSCC patients aged 75 years or more and treated between January 2002 and August 2018 by free flaps reconstruction after major OSCC procedures.

Post-operative complications were analyzed and classified according to the following criteria: (1) based on the subsequent treatment strategy, each complication was named as surgical, if it required a surgical solution, or medical, if suitable for non-surgical management; (2) based on the severity, according to the Clavien-Dindo scale (10), each complication was arbitrarily categorized as minor (grade 1–3a) or major (grade 3b−5) (Table 1). Therefore, four sets of possible clinical scenarios were considered: minor surgical, major surgical, minor medical, and major medical complications. The rate of each was then calculated and association between medical and surgical complication rates evaluated by the chi-squared test.

**Table 1:** The Clavien-Dindo classification system for medical and surgical complications.

**Grade**	**Description**	**Number of patients (%)**
Grade 1	Any deviation from the normal post-operative course not requiring surgical, endoscopic, or radiological intervention. Allowed therapeutic regimens are: drugs as anti-emetics, antipyretics, analgesics, diuretics, electrolytes, and physiotherapy. This grade also includes wound infections opened at the bedside	39 (46.4)
Grade 2	Requiring pharmacological treatment with drugs other than such allowed for Grade I complications. Blood transfusions and total parenteral nutrition are also included	12 (14.2)
Grade 3	Complications requiring surgical, endoscopic, or radiological intervention. They are distinguished into: Grade 3a—intervention not under general anesthetic Grade 3b—intervention under general anesthetic	24 (28.5)
Grade 4	Life-threatening complications. This includes central nervous system complications which require intensive care. They are distinguished into: Grade 4a–single-organ dysfunction (including dialysis) Grade 4b–multi-organ dysfunction	5 (5.9)
Grade 5	Death	4 (4.7)

Demographic, clinical, and surgical data of patients, including age, gender, body mass index (BMI), chronic comorbidities, pre- and post-operative hematocrit (Hct), hemoglobin (Hb), albumin, previous head, and neck treatments, tumor subsite and stage, duration of general anesthesia (DGA), type of surgical defect, type of free flap used, and flap outcome were collected. Moreover, the American Society of Anesthesiologists (ASA) score (Table 2) (11) and the 27-item adult comorbidity evaluation (ACE-27) scale (12, 13) were retrieved for each patient (Table 3).

**Table 2:** Patient stratification according to the American Society of Anesthesiologist (ASA) physical status classification system.

**ASA Score**	**Condition**	**Number of patients (%)**
1	Healthy	0
2	Mild systemic disease	21 (25)
3	Severe systemic disease	56 (66.6)
4	Severe systemic disease which is a constant threat to life	7 (8.3)
5	A moribund person who is not expected to survive without the operation	0
6	A person declared brain-dead whose organs are being removed for donor purposes	0

**Table 3:** Patient stratification according to the ACE-27 scoring system.

**ACE-27 score**	**Condition**	**Number of patients (%)**
0	No comorbidity	12 (14.2)
1	Mild comorbidity	20 (23.8)
2	Moderate comorbidity	45 (53.5)
3	Severe comorbidity	7 (8.3)

Once data were collected, the subgroups of “older old” and “oldest old” were studied in order to compare outcomes.

Ethical committee approval was not deemed necessary due to the retrospective nature of the study. Patients routinely signed a form consenting to use of personal data for scientific and research purposes before enrollment in any kind of diagnostic and therapeutic pathway at all three institutions.

### Statistical Analysis

A univariate analysis of factors potentially affecting the complication rate was performed using demographic and clinical information with the Mann-Whitney test for continuous variables and the Chi-square or Fisher's exact test for categorical variables, as appropriate. Level of significance for selection of predictors was set at 0.15. A multivariate logistic regression analysis with overall rate of complications (dependent variable) and factors applying significance at univariate analysis (independent variables) was run to identify predictors that are independently associated with an increased rate of complications. The test was repeated for each type of complication with a level of significance set at 0.05. *P*-values between 0.05 and 0.10 were considered to be close-to-significance.

## Results

The study included 84 patients, 48 males, and 36 females. Mean age was 79.9 years (median, 79; range, 75–90). Seventy-two patients (85.7%) were considered “older old” and 12 (14.3%) “oldest old.” Mean BMI was 24.6 m/kg^2^ (median, 25; range, 15.4–31.2). Eighteen (21.4%) patients had type two diabetes mellitus (DM). Twenty-one (25%) had a recurrent tumor and had previously received oncologic treatment (Table 4). All patients underwent free flap reconstruction. Three required two simultaneous free flaps (fibula and forearm in two, and fibula and antero-lateral thigh in one) (Table 5).

**Table 4:** Demographic and clinical data of the present series of patients.

**Variable**	**Category**	**Value**
Age (n)	75–85	72 (85.7%)
	>85	12 (14.2%)
Gender (M/F)		48/36
BMI (m/kg^2^)		Mean, 24.6; median, 25; range, 15.4–31.2
Comorbidities (%)	Hypertension	53 (63%)
	Hyperlipidemia	14 (16.6%)
	Hepatic Disease	9 (10.7%)
	Cardiac Disease	16 (19%)
	Diabetes Mellitus	18 (21.4%)
	Pulmonary Disease	12 (14.2%)
	Peripheral Vascular Disease	5 (5.9%)
	Renal Insufficiency	8 (9.5%)
Hematocrit (%)	Pre-operative	Mean, 40.59 (range, 31.10–50.90)
	Post-operative	Mean, 33.14 (range, 42.60–22.70)
Hemoglobin (g/dl)	Pre-operative	Mean, 13.36 (range, 9.7–16.9)
	Post-operative	Mean, 10.99 (range, 7.9–13.8)
Albumin (g/dl)		Mean, 4.05 (range, 3.14–5.01)
Previous RT (%)		12/84 (14.2%)
Smoking (%)		32/84 (38%)
Alcohol (%)		40/84 (47.6%)
DGA (minutes)		Mean, 553.5 (range, 230–890)
Patients requiring blood transfusion (%)		33/84 (39.2%)

**Table 5:** TNM staging and surgical data of the present series of patients.

**Variable**	**Category**	**Number of patients (%)**
T	1	2 (2.3)
	2	14 (16.6)
	3	22 (26.1)
	4	46 (54.7)
N	0	48 (57.1)
	1	11 (13)
	2	19 (22.6)
	3	6 (7.1)
Tumor site of origin	Tongue	33 (39.3)
	Buccal mucosa	14 (16.7)
	Floor of mouth	15 (17.8)
	Lower gum	9 (10.8)
	Retromolar trigone	7 (8.3)
	Hard palate + upper gum	6 (7.1)
Type of surgical defect	Soft tissues only	56 (66.7)
	Soft tissues and bone	28 (33.3)
Free flap used^*^	Radial forearm	41 (48.8)
	ALT	31 (36.9)
	LD	5 (5.9)
	Fibula	6 (7.1)
	Scapula	3 (3.5)
	Rectus abdominis	1 (1.1)

* *Multiple flaps were used for reconstruction in three cases*.

The flap success rate was 94.1%: complete necrosis was observed in five patients (two arterial and three venous insufficiencies). No rescue microvascular revision was possible and/or successful. These patients were therefore, secondarily rescued as follows: one with Bernard-Von Burrow local flap, two by pedicled pectoralis major flap, one with a further antero-lateral thigh, and one with an obturator prosthesis. Partial flap necrosis was observed in four (4.7%) patients; of these, three received additional surgical interventions (two pedicled pectoralis major flap and one abdominal fat graft for salivary fistula/cervical suture dehiscence), and one conservative non-surgical intervention. Perioperative deaths occurred in four cases (4.8%), all secondary to septic shock with multi-organ failure.

Mean hospitalization time was 23.7 days (range, 8–131). Mean time for tracheotomy and naso-gastric feeding tube removal was 10.4 (range, 3–40) and 16.4 days (range, 2–41), respectively. The majority of patients were categorized as ASA 3 or 4 (75%) or ACE-27 score 2 or 3 (61.9%). While 75% of patients presented a grade 1–3a complication according to the Clavien-Dindo scale, 25% presented a complication grade 3b or higher, thus requiring some kind of intervention.

### Overall Complications Rate

**Figure 1:**
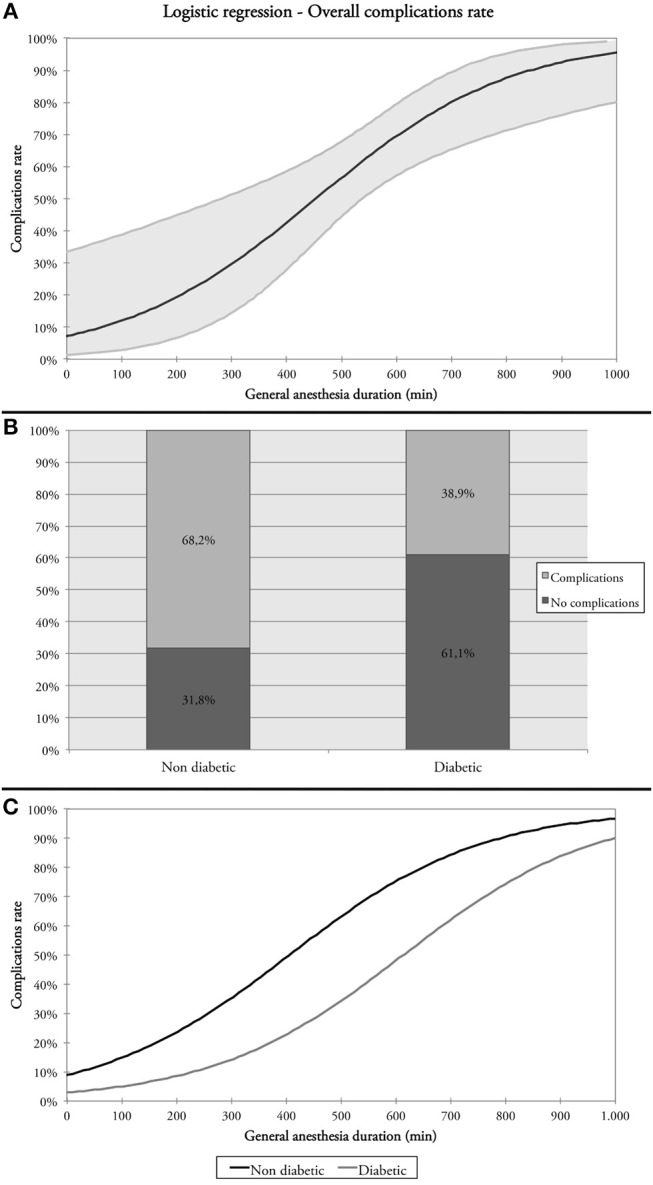
**(A)** Relationship between overall complication rate and duration of general anesthesia (DGA); **(B)** overall complication rates in diabetic and non-diabetic patients; **(C)** overall complications rate and DGA in diabetic and non-diabetic patients.

Overall, 52 (61.9%) patients had at least one complication. Factors eligible for multivariate analysis were: age, DM, post-operative Hb, post-operative Hct, and DGA. At multivariate analysis, DM and DGA were statistically significant (Table 6 and Figures 1A–C). Twenty-one (25%) patients experienced both medical and surgical complications. Such association turned out to be statistically significant (*p* = 0.018) at the chi-squared test. As a matter of fact, 52.5% of patients presenting at least one medical complication showed also a surgical one, while those without medical complications presented surgical issues in 27.3% of cases.

**Table 6:** Major and minor complications.

**Minor medical complications**	**Number of patients (%)**
Cardiovascular	11 (13)
Tracheal/bronchopulmonary	16 (19)
Hepatic/pancreatic/gastroenteric	4 (5)
Electrolytic disorders	10 (12)
Nutritional deficiency	3 (4)
Post-operative delirium	3 (4)
**Major medical complications**	
Cardiovascular	3 (4)
Tracheal/bronchopulmonary	4 (5)
Gastroenteric	1 (1)
Sepsis	2 (2)
Cerebral ischemia	1 (1)
**Minor surgical complications**	
Cervical hematoma	3 (4)
Donor site hematoma	1 (5)
Flap venous congestion	2 (2)
Flap suture dehiscence	7 (8)
Cervical suture dehiscence	2 (2)
Donor site dehiscence	2 (2)
Chylous leak	2 (2)
**Major surgical complications**	
Hemorrhage	2 (2)
Flap necrosis	9 (11)
Mandibular plate exposure	2 (2)
Fistula	3 (4)

### Minor Medical Complications Rate

**Figure 2:**
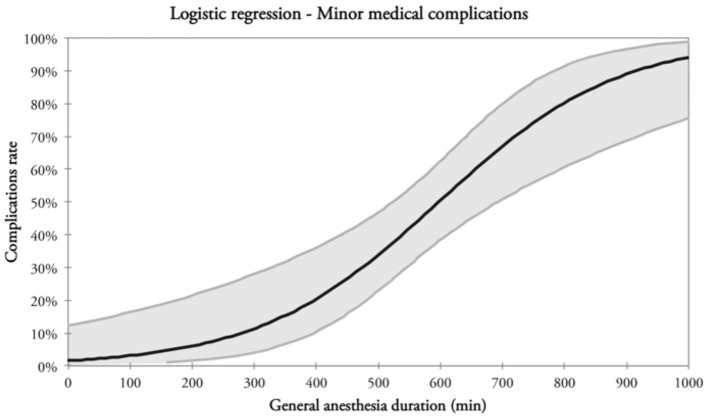
Correlation between minor medical complications and DGA.

Thirty-seven (44%) patients had minor medical complications (Table 6). Factors eligible for multivariate analysis were: age, post-operative Hb, post-operative Hct, and DGA. The latter was the only factor with a statistical significance at multivariate analysis (Table 6 and Figure 2).

### Major Medical Complications Rate

**Figure 3:**
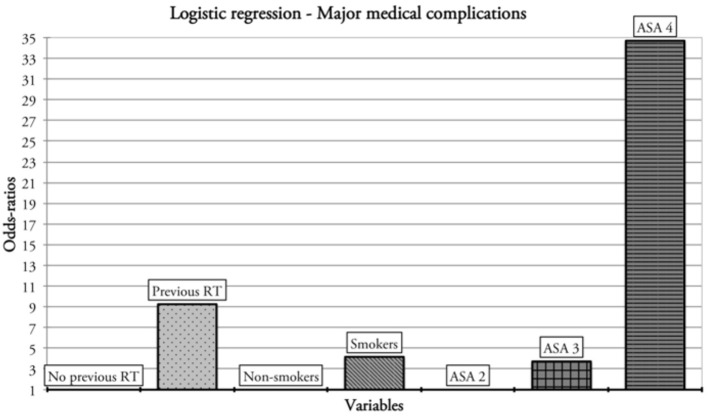
Factors affecting the rate of major medical complications.

Nine (10.7%) patients had major medical complications (Table 6). Previous RT, smoking habit, and ASA category were the factors considered eligible for multivariate analysis. History of previous RT was independently associated with the probability of major medical complications at multivariate analysis. Smoking and ASA four category showed close-to-significance *p*-values (Table 6 and Figure 3).

### Minor Surgical Complications Rate

Eighteen (21.4%) patients had minor surgical complications (Table 6). Factors eligible for multivariate analysis were: age, pT category, nodal status, and pre-operative Hb. None of these factors were significantly associated with the rate of minor surgical complications at multivariate analysis; however, nodal status was close-to-significant (Table 6).

### Major Surgical Complications Rate

Seventeen (20.2%) patients had major surgical complications (Table 6). Age, DM, tumor subsite, and type of surgical defect (bony vs. soft tissues only) were considered eligible for multivariate analysis. Age and type of defect resulted close-to-significant as potential predictors at multivariate analysis (Table 7).

**Table 7:** Results of multivariate analysis of factors independently predicting the probability of overall, minor medical, major medical, minor surgical, and major surgical complications.

**Complication**	**Variable**	**Complications rate**	**Univariate *p*-value**	**Multivariate *p*-value**	**OR (95% CI)**
Overall	DM	Non-diabetic pts: 68.2% Diabetic pts: 38.9%	**0.023**^†^	0.043^**^	0.304 (0.096–0.963)
Overall	DGA	Pts without complications: 480 min Pts with complications: 598 min	0.001^*^	0.002^**^	1.006 (1.002–1.009)
Minor medical	DGA	Pts without complications: 494 min Pts with complications: 629 min	0.001^*^	0.0004^**^	1.008 (1.004–1.013)
Major medical	Previous RT	No previous RT: 8.3% Previous RT: 25%	0.121^‡^	0.021^**^	9.253 (1.394–61.427)
Major medical	Smoking habit	Non-smokers: 5.9% Smokers: 18.2%	0.145^‡^	0.058^**^	6.146 (0.942–40.075)
Major medical	ASA category	ASA 1–3: 7.8% ASA 4: 42.9%	**0.010^‡^**	0.052^**^	25.574 (0.972–672.920)
Minor surgical	Nodal status	N0: 14.9% N+: 30.6%	0.086^†^	0.055^**^	2.997 (0.978–9.187)
Major surgical	Age	Pts without complications: 80.3 years Pts with complications: 78.3 years	0.047^*^	0.096^**^	0.877 (0.752–1.024)
Major surgical	Type of defect	Soft tissues only: 14.3% Soft tissues and bone: 32.1%	0.055^†^	0.071^**^	2.794 (0.916–8.525)

*CI, Confidence interval; OR, Odds ratio*.

* *Mann-Whitney test*.

** *Logistic regression test*.

† *Chi-square test*.

‡ *Fisher's exact test. Bold values are for statistical significant values*.

## Discussion

Life expectancy is increasing worldwide, and at a faster pace in the developing countries, not only due to improved medical and surgical treatments, but mostly for the progressively ameliorating overall social and economic status. This has led to an increase of the percentage of elderly people in the general population, thus resulting in an imbalance between advanced age individuals and their younger counterpart (14–17). In the past, the elderly were generally considered as frail subjects based on “chronological” age, and therefore major surgical treatments were mostly avoided. Recently, the awareness that “chronological” age does not reliably mirror “biological” age has emerged, thus leading to a consequent reassessment of the decision-making process in this cohort of patients (18).

Moreover, despite considerable improvements in therapeutic approaches and survival in HNC, 5-year overall survival for OSCC still ranges between 50 and 60% and has not substantially changed in the last decade (19–21). The mainstay of treatment for these tumors is upfront surgery followed by adjuvant (C)RT for advanced stages, with sound reconstruction procedures being often required to guarantee important functions such as speech and swallowing. Nowadays, free flaps are considered the best reconstructive option to restore oral cavity morphological and functional properties for two main reasons: (1) the wide range of possibilities they offer to tailor the site, type, and size of donor tissues according to the characteristics of the recipient site, and (2) the availability of highly vascularized tissues, which are frequently less involved by atherosclerotic changes, in order to speed up the healing process, especially in unfavorable conditions such as a post-actinic setting or adverse chemical conditions due to direct and massive saliva exposure. Furthermore, some complications (e.g., salivary fistula and serious bleeding) can lead to dismal outcomes, considerably lengthening the post-operative course, and should be therefore, prevented by liberal use of the most appropriate reconstructive techniques (22).

Based on these premises, there is a growing need to collect data on elderly HNC patients undergoing complex surgical procedures. Indeed, several papers on the use of free flaps for HNC in the elderly population have been published during the last 10 years. All conclude that advanced age is not a contraindication *per se* to microvascular reconstruction, and surgical outcomes in this subset of patients are comparable to those in the younger population, even though an increased rate of medical complications can be reasonably expected (23–28).

The aim of this multi-institutional study was to measure outcomes and shed light on some possible risk factors affecting elderly patients receiving free flap reconstruction for purely OSCC ablation. In our series, more than 60% of patients experienced at least one complication, and DM and DGA were found to be the most important factors affecting this event at multivariate analysis. In particular, DM affected 21.4% of our patients and, surprisingly, our findings showed that non-diabetic patients presented a higher rate of overall complications compared to their diabetic counterparts (68.2 vs. 38.9%). On the basis of well-known physiopathological concepts, it is generally accepted that DM has a negative impact on free flap reconstruction due to its detrimental effect on blood microcirculation (29). Recently, Liu et al. (30) published their experience comparing 105 diabetic to 204 non-diabetic patients (≥60 years of age) who underwent free flap reconstruction for OSCC. Their findings showed an overall incidence of flap complications of 24.3% (41.9% in diabetic vs. 15.2% in non-diabetic with an odds-ratio [OR] of 3.413, *p* ≤ 0.001) and 13.9% of major complications requiring surgical procedures (22.9% in diabetic vs. 9.3% in non-diabetic; *p* ≤ 0.001). Interestingly, vessel thrombosis (especially of the vein) occurred with a higher percentage in the diabetic group, particularly within the first 4 days after surgery (30). Other studies assessing the association of DM with the rate of complications and flap outcomes have reported controversial results, thus preventing firm conclusions (31–36). Interestingly, DM was mostly associated with flap-related complications (i.e., flap necrosis, fistula, dehiscence, wound infection) (30, 35–37) whereas rarely with systemic problems (33) which are, instead, highly represented in the present series. Moreover, the paradoxical role of DM observed in the present series might be due to the specific perioperative protocol that was delivered to diabetic patients at our Institutes. In fact, all patients with DM were comprehensively evaluated by Internal Medicine Unit staff with the following aims: (1) to prescribe tailored perioperative, continued intravenous administration of 5% glucose solution, KCl, and short- and long-acting insulin (adjusted based on periodic measurement of the capillary glucose), and (2) to assess and correct metabolic alterations typically found in such patients. This protocol might have kept patients adequately hydrated and with pre-operative glucose blood level in a normal range. In fact, a recent paper from Bollig et al. demonstrated that perioperative hyperglycemia is a common finding and is significantly associated with the overall complications rate regardless of previous DM history and management (38). This piece of evidence aligns with our findings, suggesting that in patients receiving microvascular reconstruction after OSCC ablation adequate control of perioperative glycemia can have a positive impact on the risk of complications counterbalancing the negative effects of a diagnosis of DM.

Duration of general anesthesia was an important variable in predicting complications and outcomes in this subset of patients. Our results showed that >500 min of DGA was associated with more overall and minor medical complications (480 vs. 598 min, *p* < 0.001 and 494 vs. 629 min; *p* < 0.001, respectively). This relationship was also observed by Moorthy et al. who showed that DGA had a significantly negative impact on both the rate of complications (*p* < 0.006) and length of hospitalization (*p* < 0.001) in a series of 157 patients (≥65 years of age) surgically treated for HNC. These data reinforce the belief that a double-team approach, when feasible, is a wise option to reduce the length of surgery, especially in elderly HNC patients (39).

Comorbidity assessment is crucial in the elderly and may predict both outcomes and possible complications. In our study, comorbidities were assessed through two different classifications: ASA and ACE-27 scores. The first is used worldwide to define the anesthesiology/surgical risk by virtue of its reliability and simple rating system, while the second is a more complex score based on 27 items assessing several aspects. Our findings, as expected, showed that the large majority of patients presented moderate/severe comorbidity status after evaluation by both ASA and ACE-27 scores. Moreover, the ASA predicted major medical complications with close to significance *p-*value at multivariate analysis. These data are similar to those present in the literature, confirming that high ASA scores in the elderly predict an increased risk of medical complications, without affecting either flap outcomes or perioperative mortality (23–25, 40). The main drawback concerning ASA is its non-negligible inter-observer variability that can potentially lead to important assessment biases (41). On the other hand, ACE-27 did not affect any of the outcomes considered in the present study. This is possibly due to the complexity of this scale and the relatively small number of patients considered herein. In fact, Hwang et al. recently concluded that high ACE-27 and ASA scores have very similar predictability on flap survival in the elderly (OR 5.854 and 4.397, respectively; *p* < 0.05) (42).

Interestingly, our data did not show any statistical difference in patients aged >85 years (“oldest old”) from the 75–85 year-old counterparts (“older old”). This confirms data published in the literature demonstrating that chronological age by itself can be a confounder in clinical decision making for planning treatment. However, the systematic use of a comprehensive geriatric assessment to identify patients with a high comorbidity status and advanced age (the “frail elderly”) cannot be overemphasized.

In our series, pre-operative RT was found to significantly affect the rate of major medical complications, with a 9-fold increase at multivariate analysis. This is probably due to acute and late toxicities, which can also affect organs that are non-contiguous to the RT field (43). Although the effectiveness of RT in elderly is undoubted (44, 45), Herle et al. published a meta-analysis of 24 studies on the overall effects of (C)RT on microvascular flaps, specifically focusing on its clinical impact on reconstructive outcomes. The authors compared 2,842 free flaps in irradiated vs. 3,491 in non-irradiated patients, and found a significantly higher chance of flap failure (relative risk [RR] 1.48, *p* = 0.003), flap complication (RR 1.84, *p* < 0.001), and reoperation rate (RR 2.06, *p* < 0.001) in the former, whereas no clear association with advanced age was demonstrated (46).

## Conclusions

Appropriate multidisciplinary treatment of the elderly is unavoidably becoming a hot topic in the head and neck oncology arena. In particular, advanced OSCC frequently requires extensive surgical ablation followed by potentially complex reconstructive procedures, for which free flaps are gradually emerging as the first-choice option. Indeed, apart from a number of considerations around the possibility to achieve genuine oncological radicality, there is no sense in performing such extensive resections in subjects with a presumed short life expectancy if not trying to restore the best possible quality of residual life. Therefore, adequate reconstructive technique should be always aimed to restore speech and swallowing while minimizing the risk of complications. On the other hand, lengthy DGA and in-hospital stay should be carefully considered especially when dealing with advanced age patients. However, our data herein confirm the safety, effectiveness, and reproducibility of microsurgical free flaps in patients with >75 years of age treated for OSCC. Moreover, our results suggest that DM should not be considered a risk factor *per se* in this subset of patients; rather, the attention and perioperative management should be oriented toward an optimal control of perioperative glycemia. DGA was confirmed to be a pivotal factor in determining the overall rate of complications. Therefore, surgical teams should be encouraged to reduce the duration of surgery by operating with ablative and reconstruction teams simultaneously. Finally, pre-operative comorbidity assessment by ASA and ACE-27 is effective, but should be always accompanied by mindful consideration of previous RT and smoking history.

## Data Availability

All datasets generated for this study are included in the manuscript and/or the supplementary files.

## Author Contributions

NM, DL, AV, FB, and MF: data collection and statistical analysis. AG, CP, RP, and PN: data interpretation and writing. All authors contributed to the revision of the manuscript.

### Conflict of Interest Statement

The authors declare that the research was conducted in the absence of any commercial or financial relationships that could be construed as a potential conflict of interest.
